# Speed as an expression and texture of space: theory at play in a movement activity

**DOI:** 10.1177/26349825241263982

**Published:** 2024-07-25

**Authors:** Gavin J. Andrews, Meridith Griffin, Cassandra Phoenix

**Affiliations:** 1 McMaster University, Canada; 2 Durham University, UK

**Keywords:** speed, space, movement activity, cycling, sport, health, geography

## Abstract

In recent years, following new materialist, posthumanist and non-representational turns, human geography has increasingly understood the worlds it studies as vital, immediate and emergent. As part of this vision, studies have empirically animated and theoretically articulated various expressions/textures in the movement of space, including its rhythms, shapes, timings, repetitions, sensuousness, and infections. Speed is one such expression/texture that has received some empirical attention but, in comparison to most others, has not been so thoroughly theorized. In response, this paper conducts a reconnaissance into speed, its intentions being to convey some foundational theoretical understandings of speed and, through empirical research, show these at play in social contexts. Specifically, naturalistic participant observations of forms of the movement activity of cycling are used to animate how; (i) speed can be represented and affective as a scalar quantity; (ii) all objects possess speeds and affect other speeds; (iii) speeds and objects are known through relative positions and speeds; (iv) speeds create rates of happening; (v) speeds occur in all expressions/textures of space; (vi) the accelerating world is engaged at relational speeds. From this reconnaissance, to assist future research on speed, the paper closes with some suggested avenues for further inquiry.

## Introduction: space in the new human geography

“How can… [academics] engage with speed as a processual matter that permeates our theoretical and descriptive accounts of practices, processes, and realities?” (Duclos et al. 2017:1)

This is a paper about speed; how speed is involved processually in the making of worlds and, as Duclos et al ponder, how it might be better integrated into academic accounts of them. Specifically, we come at this through a geographical lens, asking what could greater attention to speed offer an emergent understanding of space - its materiality and expressions/textures? Before we attempt to answer this latter question however, we need to provide some context and think about how understandings of space have changed and developed over time.

The new human geography holds a view of the world as vital, immediate, moving and emergent. A view of the world created, not only through humans thinking, but just as much by humans and nonhumans relating and performing. As is well documented, it is a view broadly contributed to by key theoretical traditions including non-representational theory, new materialisms and posthumanism (Anderson, 2014; Anderson and Tolia-Kelly, 2004; Castree and Nash, 2006; Falcon, 2023; Thrift, 2008; Williams, 2019) and related conceptual fields including affect theory, assemblage theory and relational theory (Anderson et al 2012; Anderson, 2014). These traditions have been responsible for diverse studies focused on the more-than-human and more-than-representational in life, but they have also contributed to the rethinking of some central concepts that frame and explain empirical realities and assist academic conversations. Some of these re-thought concepts have been general in character, including representation, agency, practice, subjectivity and the subject (Dewsbury 2000; Glass and Rose-Redwood, 2014; Nash 2000; Simpson, 2017a; Rose, 1999; Wylie, 2010), whilst others have been geographical in character, including place, scale, distance and world (McCormack, 2017; Marston et al 2005; Merriman, 2023 Simandan, 2016). Of the latter group, notably space has been prominent, it the subject of significant ontological discussion, extension and reform (Doel, 1996; 2000; Jones, 2009, Kitchin and Dodge, 2005; Malpas, 2012; Massey, 2005; Merriman, 2012; Merriman et al 2012).

Some longstanding visions of space, although possessing long philosophical lineages, have been questioned in recent years by the new human geography for their assumptions and scope. Table 1, summarises five examples; Newtonian, Euclidean (see Merriman, 2022; Meyer, 2024), Marxist (see Harvey 1982; 2001; Sheppard and Barnes, 1990), phenomenological (see Pickles, 1985; Buttimer, 1976), and a particular poststructuralist vision (see Philo 2012; Thrift 2007). Notably, the mechanisms in the production of space common to such visions, have not necessarily been rejected by the new human geography (whether, for example, they be location, capital, cognition or power); they are still recognised as important. But there has been an acknowledgement that they are each active in less discrete, exclusive and all-encompassing senses, and that one needs to think about what goes on ‘prior’ to them in space physically, materially and processually, to create them in their different forms. Overall then, the new human geography is less concerned with the cause or representation of space, and is more concerned with fundamental processes of space.

A number of understandings have emerged in the new human geography that together install an underlying metaphysics that holds a vision of space as materially contingent and constituted (see table 2 for an overview). At their core these understandings broadly concur with the idea of space emerging initially, not through human subjectivity or external transcendental power or design, or with set teleological propensity, but through a self-organizing open process of mattering. This occurring within what Deleuze and Guattari (1988; 1994) have famously termed the ‘plane of immanence’; a preconceptual abstraction describing virtual relations necessary for actual events and concepts (tribal/intensive assemblages). The plane of immanence an unstructured, neutral and ongoing field, within which all type distinctions are flattened (all elements possessing positions, proportions, orderings, directions and relations). The idea that space is ‘pure’ variation in this plane - created by the endless knotting of objects, affects and cognitions that emerge within it - is a helpful theoretical starting point and visionary. In sum, being mindful not to oversimplify these understandings of space in the new human geography, and not to overlook the complexity, contested nature and long history of the theory from which they are derived (see, for example, arguments of geographers (Anderson et al 2012; Doel, 1996; 2000; Jones, 2009; Malpas, 2012; Massey, 2005; Merriman, 2012; Thrift, 2003; 2008) and social theorists (Barad, 2007; DaLanda, 2016; Harman, 2018; Leibniz, 1956; Nail, 2019; Pred, 2005)), they add up to a realization that space is not a cause or outcome of things, rather it is part of all things.

With regard to humans in particular, without delving into extensive debates here on the nature of subjectivity and being, space is recognised in the new human geography as much more than something noticed by us, or something that we participate in or contributed to. Just as we help make it, it makes us. Indeed, a (Merleau) Pontian and later posthumanist reading, is that with our continually moving sensing, interacting bodies, we are not just *in* space, we are *of* space; produced in all our character through such relations (Merriman, 2022) (or we are *of* the plane of immanence; our being merely a point on it). Yet of course we do still experience space, and this aspect is important in overall processes. A Thriftian reading is that we register various forms and configurations of material space (as mentioned at the bottom of table two) with various degrees of consciousness, and that their consistency gives our worlds coherence and us a basis for further performative engagement in them (Thrift, 2008). Moreover, a Predian reading (later endorsed by Thrift) is that the changes in forms of space we are exposed to, sense and engage over time – arising through our movements and interactions and the movements and interactions of objects in our vicinities – provide one basis for our registering of our personal ‘onflow’. This is our seamless, unbroken steam of experience, including its feeling of ongoingness. Our experience of onflow – and the interplay of perception and action it involves - being where and how our consciousness is generated (Pred, 2005; Thrift, 2008).

It is what has proceeded from this rethinking of space, that has given much of the new human geography a quite specific empirical knowledge-base, shaping its distinct character and reputation. That being examination of the vital, material, registerable, ‘expressions/textures’ in the movement of space(time) as it unfolds as the progressing moment (albeit acknowledging that spacetime itself has a broader history of application (Janelle, 1968; Isard and Liossatos, 1975), refinement (Massey, 1992; 2005) and critique (Andrews and Duff, 2020a; May and Thrift, 2001; Merriman, 2012)). The most widely discussed of these material expressions/textures have been: (i) *The predominant flows, rhythms and momentums of space* that enroll and carry life forward (see Edensor, 2010; 2012; McCormack, 2002; Mels, 2016). (ii) *The positionings, spacings, shapes and lines of action of space* – many performative - that lend it basic expression (see Doel, 2000; McCormack, 2005; 2013; Schwanen, 2007). (iii) *The imminences, events and encounters of space* that create points of change in it and the world (see Laurier and Philo, 2006; Shaw, 2012; Wilson, 2017). (iv) *The sensuous properties of space* that make it recognisable and ‘thick’ to entities registering it (see Kanngieser, 2012; Paiva, 2018; Paterson, 2009; Rodaway, 2002). (v) *The habitual or machinic, often synchronised, forms of repetition in space* that give it certain character and predictability (see Bridge, 2020; Dewsbury and Bissell, 2015; Schwanen et al 2012). (vi) *The affects at work in space* – i.e. the spatial dance of bodies and objects affecting one another and increasing in potential and energy – that gives space particular somatic feel, intensity, vitality and infectiousness (see Anderson, 2014; Bissell et al 2017; Pile, 2010; Thrift, 2003).

The diverse intellectual underpinnings and operational specificities of these six expressions/textures acknowledged (notably Badiou, 2005; Deleuze and Guattari, 1988; Lefebvre, 2004; Massumi, 2002), there seems to be some level of consensus in the literature on what collectively they are, what they do and how they are registered, including that: (i) They occur in and amongst entities *in space*. But, because space is the collective relational accomplishment of entities, they also become expressions *of space* itself. (ii) They are simultaneously structural components of space (i.e. necessary functional parts of it and its productions), and qualities of space (i.e. expressed and registered as particular amplifications of it). (iii) They are more-than-representational, in that they are non-textual, non-verbal and physical - initially performed, registered and felt in ways prior to words and meaning (hence they are an important part of the practice of life and its registers). (iv) They are a critical, vital, energetic ingredients in the world’s complex productions (i.e. in all objects and systems, and the processes, transformations and outcomes they involve). (v) They are general in character but are unique to each occasion. This meaning that they transition over time, and that each entity exposed to them possesses unique spatial positions, involvements, capacities and registers in relation to them. (vi) They are all always simultaneously present and concurrently occurring, together they creating the overall expression and texture of progressing space(time). (although in particular contexts and moments, certain ones might combine in mutually supportive manors and/or come to the fore over others in terms of roles and registers). (vii) They are ever prominent in the current posthuman era characterised by the emergence of diverse technological systems and processes which create new forms of activity, movement and artificial atmospherics.

Despite this broad coverage of the material expressions/textures of space, there is relative lack of theoretical attention in the new human geography directly and specifically to speed as one; a potential seventh (Andrews et al 2022). As will be noted, speed certainly comes through to different extents in various empirical research, yet this theoretical gap is perhaps surprising for two reasons. First, given general theoretical attention to speed in cognate fields - such as cultural theory (Virilio, 1986; 1995; 2005) and sociology (Rosa, 2003; 2005; 2010) – that often inspire and inform geographers. Second, because of the immutable fact that, regardless of how significant speed might be as an expression/texture of space in itself, all other expressions/textures involve movement, so all must necessarily involve speed. It is this theoretical gap that the current paper seeks to address. We want to undertake a reconnaissance into the subject by finding and describing some foundational theoretical understandings of speed as articulated across the sciences, social theory/philosophy and the social sciences, and present empirical data to animate these at play in a particular socio-spatial context (the nature of this search and data we will describe shortly). This reconnaissance, we hope, will potentially help inform future studies of speed. And, also to this end, we will close by providing some final thoughts on some specific avenues for future inquiry.

## Speed in empirical considerations in human geography

Before we proceed to our theoretical reconnaissance into speed, we extend our focus on speed beyond theories of space, to how speed arises more generally and more substantively in human geography. In one sense speed arises across human geography as a measure, variable and quality of empirical phenomenon. Indeed, it is used in describing the movement of empirical phenomenon, or the relationship between empirical phenomenon, through time and space. As table 3 shows, speed is attributed varying degrees of priority, of sophistication and of conceptual attention, ranging from its use as in common everyday language, to it being a key variable in certain fields.

Beyond such treatments, speed is however a more general empirical ‘topic’ in and of itself in human geography, where the rapid pace of early twenty-first century life (‘the great acceleration’) has emerged as a specific, broad concern (Crang, 2007; Levine, 1997; 2005; May and Thrift, 2001). It has been observed that in the current era, aided by technologies, new forms of speed and increasing speeds occur across multiple domains; in transport and mobility, in communication and networking, in arts and entertainment, in work and productivity, and in development more generally (Latham and McCormack, 2008; Merriman, 2017; Tomlinson, 2007). Speed hence becoming the primary rationale for innovation and the key barometer of social progress (Wajcman and Dodd, 2016). For speed theorist Virilio - whom is often cited by geographers - society is now ‘dromological’ (i.e. best characterised by speed); it in perpetual motion, an increasingly delocalized and intense, ever-enlarging domain of speed (Luke and Tuathail, 2002).

It has been noted that, for humans, the result of participating in such a society is an increase in the speed of their everyday lives lived. Levine (2005) conceptualizes this as an increasing ‘busyness’ (speed + activity). But, in terms of experience, it is also more than just busyness. At one level, the ever-increasing pace of life can feel – and be – debilitating or exhilarating as the future rushes towards us seemingly out of control (May and Thrift, 2001). At another level, when operationalized through such things as electronic media and communication, speed instills rapidly changing, virtualized, subliminal perceptions that exist alongside and affect slower more conscious–visions, thoughts and reflections (Luke and Tuathail, 2002). At the same time however, there is clear evidence of things that do not fit or oppose the idea of all-world acceleration. On the one hand, reacting to modernity’s ever-increasing speeds, numerous counter-movements have emerged that, through their practices, (re)instill slowness in life. These include slow retreats, slow tourism, slow holistic practices, slow food, slow homes, slow cities, slow fashion (Conradson, 2011; Crewe, 2000; DeVerteuil, 2022; Lea et al 2015; Paiva et al 2017; Raco et al 2018; Vannini, 2014; Vannini and Taggart, 2015), and even ‘slow reasoning’ and ‘slow science’ (the latter demanded in the face of environmental challenges, to resist forces that lead to pressed thought, automatic responses and suboptimal outcomes (Whatmore 2009; 2013) or more generally to improve the quality of research and teaching in speed-obsessed corporate universities (Berg and Seeber, 2016; DeVerteuil, 2022)). Although, as Luke and Tuathail (2002) note, for Virilio and others, these counter movements are merely ‘dromological experiments’ seeking to temporary reinsert traditional human agency against the dominant unchanging flow. On the other hand, another inconsistency is that many humans and places are simply excluded from faster life and worlds due to their economic or social positions (Creswell, 2010). Hence, through differential access to and exposures to it, speed is at the centre of many hierarchies in society.

In human geography, conversations on the increasing speed of the world have often been part of wider debates on time-space compression (Harvey, 1989; 1996; Thrift, 1996). As scholars of time-space compression have suggested, in recent decades greater speeds of objects (both physical and electronic/informational) have produced greater speeds of events (whether these events be communications, transactions, processes or mobilities). Thus, in overcoming a host of material frictions and limitations, and in conquering space and time, humans have been able to recalibrate relational notions such as close/near and distant/far (Warf, 2008; 2011). It is recognized that, contrary to the literal meaning of time-space compression, time and space are compressed only in secondary ways (Dodgshon, 1999). Hence, underlying clock time which governs our lives does not actually compress, rather the time taken for specific events to occur does; this facilitating new relations of transformation and succession. Likewise, overall world space does not actually compress, rather more space is covered by, or incorporated into, specific events; this facilitating new relations of structure and organization (Dodgshon, 1999). Also, it is recognized that under time-space compression, time is ultimately elevated over space. Specifically, as Luke and Tuathail (2002) note, time systems and their chrono distributions replace space and spatial distributions. And real spaces – national and international – give way to communications in ‘chrono-proximity’. Critiques have been mounted of general accounts of the time-space compression, they calling for an understanding of its limits and downsides (May and Thrift, 2001 and see table 4). In sum though, through such critiques, there has been an acknowledgement that time-space-compression involves multiple entities in multiple roles and positions, multiple insiders and outsiders, and entangled ecologies and practices (Massey, 1994; Searle et al., 2021).

Attention has been paid to establishing and explaining what might be the primary engine and driving force of the increasing speed of the world. As Luke and Tuathail (2002) note, such explanations are largely focused on either the ‘means of destruction’ or the ‘means of production’. With regard to the former, Virilio and others have considered the engine to be military hardware accumulation (that redefines how space might be dominated, crossed, captured and retained). This accumulation involves faster information, faster weapons, faster taking of territories, and faster completion of military campaigns. The advancements that have facilitated these new speeds, they argue, have tricked-down and affected everyday civilian technologies and life. With regard to the latter, Harvey and early Marxist geographers considered the engine of the increasing speed of the world to be capital accumulation (they highlighting ideas on accelerating production and circulation in Marx’s original work). More recently, along with other social scientists, geographers have refined early Marxist explanations, describing the engine as a new expression of capitalism; specifically what has been termed ‘fast capitalism’ (Agger, 2015; Andrews and Duff, 2020b). Indeed table 5 outlines four defining features of fast capitalism in the current era that have led to more rapid, efficient and widespread production and consumption, in increasingly dense patterns and entanglements. In short, fast capitalism is recognized as ever more quickly occupying, crossing, recalculating, retexturing and reengineering space (Thrift, 2004; 2008).

These empirical realities are very important and suggest the need for geographers to dig deeper and engage theoretically with speed as an expression of space. Such a theoretical engagement might support both a continued empirical engagement with the faster world and a far broader empirical engagement with the speeds in all practices and productions (irrespective of whether they are moving faster or slower in the current era). Indeed, practices and productions always involve speed. As Thrift (2008:5) notes ‘all life is based on and in movement…. [it] captures the animic flux of life and especially an ontogenesis which undoes a dependence on the preformed subject; every creature, as it issues forth and trails behind, moves in its characteristic way’. And as Deleuze (2005: 58-59) argues, the ‘important thing… is to understand life, each individuality, not as form or a development of form but as a complex relation between differential velocities, between deceleration and acceleration of particles. A composition of speeds and slownesses on a plane of immanence’. Hence ultimately, researching speed ideally involves far more than reporting geo cultural or historical changes in the practice and experience of speed. It involves a commitment to thinking through the role of speed as a constitutive dimension to the fundamental fabric of all things (Duclos et al. 2017). It is this that we want to animate through empirical study.

## Animating speed theory

Our first task was to review current ideas on speed across sciences, social theory and social sciences. This was to establish some foundational and generally consensus theoretical understandings of speed that we could look for and animate as empirical and spatial realities. The review was conducted whilst acknowledging the potential pitfalls of drawing from different disciplines and theoretical paradigms to establish understandings of speed. Specifically acknowledging that we needed to remain aware that the wider ontological worldviews of these disciplines and paradigms do not always align, and that their understandings of speed might be arrived at and operationalized very differently. Moreover, acknowledging that we needed to remain aware not to conflate or confuse very different academic engagements with, positions of, or uses of, speed between these disciplines and paradigms (for example, speed being a tool and a thing to physics, but speed being part of describing the virtual or actual or a preconceptual abstraction in certain metaphysics). Nevertheless, the six relatively uncontroversial understandings that emerged from this extensive review can be paraphrased as; (i) speed can be represented and affective as a scalar quantity; (ii) all objects possess speeds and affect other speeds; (iii) speeds and objects are known through relative positions and speeds; (iv) speeds create rates of happening; (v) speeds exists in all textures/expressions of space; (vi) the accelerating world is engaged at relational speeds (see Andrews et al 2022). Each is explained in the following findings sections, alongside corresponding illustrative data.

In terms of finding a something to empirically illustrate these six understandings, we noted that in studying other material expressions/textures of space, geographers and others had in the past focused on physical/movement activities through mobile ethnographic methods, particularly in studies cast as sports and fitness geographies (Andrews, 2017) or positioned under the broader new mobilities paradigm (Cresswell and Merriman, 2011). The reason for this particularly strong track record is likely because these activities involve purposeful performances and movements that create, dominate and characterize spaces; this a basis for their wider reproduction and identity as physical/movement cultures. These studies can be mapped loosely onto the typology of expressions/textures of space listed earlier. With regard to flows, rhythms and momentums, work exists for example on rural running (Lorimer, 2012), mountain-stage cycling (Spinney, 2006) and night-cycling (Cook and Edensor, 2017). With regard to positionings, shapes, spacings and lines of action, work exists for example on dance (McCormack, 2013) and parkour (Ameel and Tani, 2012). With regard to imminences, events and encounters, work exists for example on kayaking (Waitt and Cook 2007), road running (Cook et al 2016) and mixed martial arts (Green, 2011). With regard to sensory properties and registers, work exists for example on mountain biking (Brown, 2017), group walking (MacPherson, 2009) and surfing (Evers, 2009). With regard to habitual and machinic forms of repetition, work exists for example on urban walking (Middleton, 2011), jogging (Latham, 2015) and golf (Bissell, 2013). And with regard to affects, work exists for example on alternative sports (Thorpe and Rinehart, 2010), roller derbys (Pavlidis and Fullagar, 2015), commuter cycling (Simpson, 2017b) and swimming (Foley, 2015).

Although speed is not often an explicit, direct or full consideration in this literature on physical/movement activities (as noted in table 3), speed emerges as part of the motions and sensations they involve and their various functional outcomes, whether the latter be therapeutic, personal, social or economic. Speed comes through for example, in the way that rural walkers proceed forward by employing specific techniques. They in consecutive ‘in-between positions’ with each step. Always in a continual process of departure and arrival; of leaving one landscape behind whilst entering another (see Edensor, 2010; 2017; Wylie, 2005). Speed comes through in the way that urban walkers have particular walking styles and conventions, and how their progress through a city is dependent on their navigation of various obstacles. They being aware of the increasing length of their time taken when they are slowed down or forced to stop (see Middleton, 2009; 2010). Speed comes through in the way that swimmers experience sea swimming as a moving, embodied, affective therapeutic practice. Each stroke propelling them forward in micro-bursts (see Foley, 2015; 2017). Speed comes through in the way that walking groups – such as for impaired people or those recovering from illness - are simultaneously assisted by collective movement and collective narrative/conversation as they proceed through natural landscapes (Ireland et al, 2019; MacPherson, 2008). Speed comes through in the way that the progress of running and cycling are contingent on the surfaces and textures sensed and crossed. On what landscape is constantly approaching, on the function and purpose of the run/ride, and on times of the day/night and season it is undertaken (see Brown, 2017; Cook and Edensor, 2017; Larson, 2018; Lorimer, 2012). Speed comes through in the way that dancers achieve particular therapeutic connections and outcomes through quickening or slowing down the specific lines of action of their bodily movements (McCormack, 2004; 2013). Finally and more broadly, speed comes through in the ways through which entire physical/movement cultures are established and assigned value, such as those associated with competitive running (Bale, 2004; Bale and Sang, 2013).

Given this precedent, we decided to consider speed also through movement activities. In the end we chose four forms of cycling; a popular study activity amongst geographers often viewed through non-representational, new materialist and posthumanist theoretical lenses (e.g. Brown and Spinney, 2010; Cook and Edensor, 2017; Jones, 2012; Simpson, 2019; Spinney, 2007; 2009; Waitt and Buchanan, 2023). These forms were indoor cycling (spinning), trail recreational cycling, mixed terrain commuter cycling, and fitness road cycling, each of which involved specific body-machine assemblages (but, rather than being selected for comparison, they were selected for the range they together offered). We realized that researching speed as an expression and texture of space would require methods to capture physical and embodied events across space and time, and to capture the finer details of practice, and that the findings we generated should possess a fidelity to these things (see Cadman, 2009; Vannini, 2015). As such, the specific method we chose was naturalistic participant observations (Angrosino, 2007) (naturalistic in that they were of things we already did in our regular lives, and were certainly less identity-focused than other forms of ethnography). These observations were undertaken by us over the spring and summer of 2022. GA conducted the observations both of recreational trail cycling and spinning. MG conducted the observations of mixed terrain commuter cycling, and CP conducted the observations of fitness road cycling.

In terms of our positionalities, four are particularly relevant. Academically, with regard to theory, GA is most emersed in the worlds of posthumanist and non-representational theory (traditions that are obviously close to the current paper), although MG and CP both increasingly explore the affective, sensory and performative dimensions of life in their scholarship. Academically, with regard to disciplines, only GA is a geographer by training interested specifically in the nature of space, although MG and CP (both critical kinesiologists/sport and exercise scientists) are interested in movement, space and time. Academically, with regard to the empirical field, physical activity/sport is the primary career interest of both CP and MG, whilst it is more of an occasional interest for GA via his contributions to sports geography. Personally, with regard to physical activity, all three researchers – GA, CP and MG – have undertaken multiple forms of physical activity throughout their lives, at levels that allowed them to compete in amateur sports (although MG and CP have certainly retained greater levels of fitness and involvement that GA as they have approached middle age). In short, with regard to theory, disciplines, empirical interests and personal circumstances, GA, MG and CP are variously insiders and outsiders, each bringing expertise where they are insiders, and fresh perspectives where they are outsiders. These combinations are seen as strengths, and are why the research/authorship team is composed as it is.

GA’s recreational trail cycling involved six rides undertaken with family members along their usual a 6 mile suburban trail. This is a short ride that they complete for both fitness and socialization reasons. The trail, completed on mountain/hybrid bikes, is a dirt or concrete track running through woodland, parks and the back of housing estates/subdivisions. It is purposefully designed to allow reasonable speeds with enough room to pass. It has a few sharp turns, a few short hills and good signage. The trail is used by cyclists, walkers and runners in various combinations, the volume largely depending on the day and prevailing weather. Meanwhile, GA’s spinning sessions were each 45 minutes long, and took place in a specialist studio on a small industrial estate, twice a week over a four-month period. In the minimalist studio, all objects are orientated to spinning – from the instructor’s elevated podium, to the futuristic leaning bikes, to the speakers, to the cooling fans. In many ways then this added up to a purposive ‘synthetic’ environment and atmosphere. The majority of the fellow spinners were women under 40 and, in contrast to GA, many appeared to have a high level of physical fitness.

MG’s mixed terrain commuter cycling involved a regular lone journey to and from work/campus (3 miles each way) at the start and end of her working day, for a four-month period. The commute took place through a variety of settings: starting out in a suburban neighbourhood, linking to a portion of an historical railway repurposed into a stone-dust covered multi-user trail, and finishing with some navigation of slightly busier urban roads and intersections on the approach to campus. The majority of MG’s commute occurred on the aforementioned trail, which is fairly flat. This trail is open to hikers, bikers, and horseback riders year-round. Most other users are dog-walkers and other cyclists in the morning, and families walking and cycling in the early evening. When closer to campus on the roads, MG negotiated pedestrians, cyclists, cars, and both city and commuter buses.

CP’s road cycling involved her cycling alone or with others, over rural routes **-** typically between 45km and 55km in length - weekly over four months. This activity was new to her (but not partaken specifically because of this study). All of the routes were circular (home to home). Most involved undulating quieter roads and single-track country lanes that pass through open countryside (agricultural farming) and former mining villages. The first and last 10 minutes of each ride involved pedaling through busier residential areas and along a main trunk road. CP’s road bike is designed for maximum speed and for efficiency on longer rides (often at the expense of comfort); its thin, smooth tyres reduce friction on the road and make the bike lighter. Its dropped handlebars offer CP the opportunity to change her body position, her center of gravity and the muscle groups she uses.

In terms of process, all three of us - GA, MG and CP - went into the data collection period with a good idea of six aforementioned theoretical understandings of speed as found in the multidisciplinary literature reviewed. This helped us focus our reflections on how they arose in our activities. As per ethnographic convention, we took detailed notes after each ride. We paid particular attention to animating non-representational physical aspects of speed (to showing what speed is, in addition to what it means). In terms of the analytical approach, although the six theoretical understandings of speed provided a structure for the findings, we still used thematic analysis to systematically identify and organize data and decide how it might be apportioned between the six (Braun and Clarke, 2021). We used regular word processing software to assist the exploration, management and evaluation of the data, and facilitate the careful serializing and integration of the material. We established the ‘credibility’ of the study findings through investigator triangulation. Meetings between us were used to review themes and minimize idiosyncratic bias. The selected field notes provided in each of the following sections are the ones that we feel provide the best animation of the ideas that directly precede them.

## Findings

### Theoretical foundation I: speed can be represented and affective as a scalar quantity

Innumerable movement exists in the world, particularly in the current era, with so much of it quantified through the use of technology. Indeed, measured speed is a common epistemological instrument of individuals and society (Andrews et al 2022); a reference point to help comprehend movements, events and their consequences. It of value in terms of both freedoms and controls, and a tool of human development (e.g. Rietveld and Shefer, 1998).

In terms of categorization, speed is known as a scalar quantity - i.e. a quantity of magnitude alone - that describes an object’s rate of motion over a distance between points (Elert, 2022). In this way speed is distinct from velocity, a vector quantity - i.e. a quantity of both magnitude and course - that describes an object’s rate of motion over a distance in one specific uninterrupted direction (less applicable in many human contexts) (Andrews et al 2022). Overall average speeds can of course be calculated for objects (speed = full distance / time taken) through distance and time measures of choice (mph, kph, mps etc), either manually/mentally or assisted by technologies such as tachymeters. This happened often in the field, where average speeds were calculated manually/mentally prior to, during or after rides, ultimately consideration of these average speeds affecting future practice CP, for example, describes a situation where her and her fellow cyclist had travelled too far from home:

**Fitness road cycling CP:** We are miles from home and short on time! Once again, my cycling buddy Susan and I have been unable to resist the temptation of incorporating a couple of additional loops into our early morning ride. We pull over to check the current time, how long it took us to get to this furthest point, and the number of minutes remaining before we each need to be home preparing for the school run. Attempting to ease our panic, Susan scrolls back through her Strava feed to find a more precise timing for the second half. “It’s just about doable”, she says “if we really motor it from here, at least 16 mph”.

Such practices are reflective of Parkes and Thrift’s (1975) early articulations in human geography of ‘timing space’ (i.e. calculating the times taken to cover set spaces at certain speeds) and ‘spacing time’ (i.e. calculating the spaces covered in set times at certain speeds). These early articulations directed attention to the ‘chronosophical’ skills and practices that humans possess and employ pertaining to time; skills that involve knowledges and assessments of speed.

In many social contexts however, overall average speed is not calculated when an indication of speed is needed because taking into account the full distances of objects’ movements is either prohibitive or not useful. Instead then, objects’ ‘instantaneous speeds’ (i.e. speeds at specific moments in time), are either calculated using technologies such as speedometers (that either calculate over very short times and distances or are calibrated to surrogates such as cogs and wheels), or are subjectively estimated (Ahn et al, 2002; Denton, 1966; Elert, 2022). Both approaches were common in the field in quite straightforward operational ways, and again they affected future practices. GA, for example, describes the emphasis placed on technology, whist MG describes her subjective assessments:

**Recreational trail cycling GA:** Instantaneous speed is important. Brother-in-law Rich has a relevant technology; a little red handlebar mounted GPS which has a speedo and helps us, as a group, keep a relatively constant speed and keep together. Overall it’s an integral part of these trail rides.

**Commuter cycling MG:** Today I paid attention to moments where I deliberately sped up or slowed down (my instantaneous speed) in reaction to the movements, speeds, and actions of others. When passing a slower cyclist, I needed to first mentally gauge their speed and adjust mine accordingly by a couple of kph. Where the trail crossed a road, I had to speed up by quite a few kph to go through a gap in the flow of traffic.

In sum, speed differs from some other textures/expressions of space (such as rhythms, certain sensuous properties or infections) in that, for constituent entities, it is more easily and frequently quantified with technologies and regulated with greater accuracy. Moreover, it is more easily and frequently mentally estimated with reference to familiar scales. Indeed, through its representation by numbers, there are numerous opportunities to know, create, maintain and change the speeds of the world.

### Theoretical foundation II: all objects possess speeds and affect other speeds

Every object in the world possesses speed because every object moves to some degree (Deleuze, 2005; Massumi, 2002). Newton’s First Law of Motion (the Inertia Law) states that an object will remain at rest/still, with no perceptible or recordable movement, unless acted upon by an external force (Pfister, 2004). However, in practice, no object is ever fully still, because external forces are ever-present in the universe, subjecting objects to mechanical input as they work to induce movement (Nail, 2021). Indeed, molecules and atoms vibrate, and their electrons occupy orbitals even at temperatures close to absolute zero (Morton, 2016). Larger objects vibrate and move as entireties because of environmental effects (these effects the collective movements of surrounding entities). Ultimately all objects also move because they are located on rotating planets (… which are rotating around stars, which are moving in rotating galaxies, that are moving further away from each other). These things happen as part of the one universal movement. Since the big bang, the entire cosmos and everything in it has been nothing but moving energy flows and matter cycles engaged in an overall process of increasing spread and increasing entropy/disorder (Nail, 2019). Even nouns (car, tree, house, dog, body, city…) are but conceptual shortcuts and abstractions for what appear to be stable and discrete entities, but are in fact temporary creations - moments in internal and external movement processes, stages in continued ordering then disordering - in the one universal movement (Nail, 2019). Stillness is only a perception by an observer of an object relational to other objects in a universally moving world (Andrews et al 2022), just as movement is itself only something relative to other movements. Locally, such constant movement was observed and felt in the field, particularly when cycling stopped. Such basic experiences of the world were often positive and resonate with Thrift’s (2008:5) contention that often ‘movement captures the joy – I will not say simple – of living as a succession of luminous or mundane instants’. MG, for example explains:

**Commuter cycling MG:** I feel effort in my body, usually in moments of forced stillness (stopped at an intersection). Those moments are when I can sense my heart beating more quickly, I can hear my breathing, my muscles first relaxing and then becoming poised to set off again. While the movement of cycling is paused (my feet on ground, the pedals stationary), there is still movement all around me – people walking, driving cars, rushing to begin their workday or to get home in time for dinner. Otherwise, wind blowing, branches swaying and cracking, birds flying – nature carrying on

Relational encounters are critical to speed. Objects coming together that possess complementary movements and speeds increase their individual and/or collective movements and speeds, and increase the profile and potential of those movements and speeds (Nail, 2019). This was reflected in the field. GA, for example, talks about the lack of resistances in/of his new bike:

**Recreational trail cycling GA.** I got a new bike last week. In contrast to my old one, the chain is running smoothly, the back brake isn’t binding, the shifter is fluent. Suffice to say, its parts work well so I can exert more effort for a given speed or simply go faster.

Likewise, objects coming together that possess counter movements and speeds decrease their individual and/or collective movements and speeds, and decrease the profile and potential of those movements and speeds (Nail, 2019). This was also reflected in the field, with CP describing her encounter with head wind:

**Fitness road cycling CP:** Today’s ride up a steep and long incline is slowed due to a gusting head wind, the effect increasing with the wind speed. I tuck in close behind Lindsay, lining my front wheel up with her back wheel to benefit from the shelter provided by her slipstream. We stop at the top to catch our breath, blow noses, adjust clothing, check tracking devices and drink. Lindsay then clicks one foot back into her pedals signaling the “pitstop” is over. I quickly pull my gloves out from my back pocket. A sweet wrapper flies out with them and is instantly swept up by the wind and blown back down the road from where we came.

Both of these relations are ever more common in the current era and world composed of many moving objects related and operating in complex systems. They are a critical part of the material events that change the world; that transform, providing new arrangements, movements, directions, intensities and productions (Deleuze, 1994; Nail, 2019).

### Theoretical foundation III: speeds and objects are known through relative positions and speeds

For Einstein (1905) and the Special Theory of Relativity, the speed of light in a vacuum is the most important constant in the universe, it proceeding all other constants and the same from wherever it is observed. Beyond this however, all object’s speeds vary according to propulsion/function (i.e. the power of entities creating them and the forces they exert), and environmental limits and affects (i.e. the power of entities opposing them and the forces they exert). In terms of humans knowing of an object’s speed, two variables are key. On the one hand, their visual, other sensory and cognitive functioning (Norman et al 2003). On the other, their relative positions to other objects witnessed (Denton, 1980). These both as speed is either pre-consciously registered and felt through their bodies, and/or consciously mentally judged. Both variables were reflected in in the field, with GA, for example, recalling a speedy ride:

**Recreational trail cycling GA:** Today I really experienced speed; the buffeting of the wind, the wheels rolling underneath me, rushing passing walkers, leaning into the bends. More than ever today I realised that speed provides a sensation, not easily put into words at the time.

When the speed of an object is felt in relation to, or consciously compared to, the speed of another object, this relationality provides the basis for judgments or feelings on past or current or future speed. This might be that something was/is/could be moving. Or that something was/is/could be moving at a particular speed. Or that something was/is/could be moving fast/faster/to fast, or slow/slower/to slow (Andrews at al 2022). This was reflected in the field, with CP for example describing her subjective experiences moving through roadworks:

**Fitness road cycling CP:** They say “The longest mile, is the last mile home”, especially on a hard ride, or when time is short. Today, a new addition – “the longest mile, is the one where there are roadworks with 2-way traffic lights, and you find yourself leading a convoy of traffic through the section of single track”. The pressure of a line of traffic crawling behind me, desperate to proceed with their journey after being held up on a red light, made my riding feel excruciatingly slow. I worried that I would not reach the end of the roadworks before the lights turned green again for the waiting line of oncoming traffic. When I do, I hear revs building behind as the convoy accelerates and one by one, the cars zoom past.

At the same time, an object’s speed is not only felt or judged in terms of its relative positions to other objects (and their movements and speeds), more generally, an object is defined in the same way. This is a contention with an established theoretical basis. Indeed, from a phenomenological standpoint, the ‘intentionality’ of objects (in simple terms, their potential use/function – what we understand they do) and their ‘essences’ (in simple terms, their qualities – what we register that gives us a feeling of, and familiarity with, them) are important parts of the overall meanings we ascribe to them. But in both cases intentionality and essences are not static, and an object’s form and speed of movement play an important part in each. In the following example, GA’s spinning is about moving in certain ways at certain speeds with other people and objects for certain ends; ways involve the registering of collective body-machine affects and aesthetics:

**Spinning GA:** Spinning is about moving quickly. In the spring I was the slowest person in the room. The ride was too fast for me and I felt, and was, separate from it, not part of the class. Now in the summer I’ve improved and I can match the speed of the other riders. I’m on the same ride “with” the others and part of the class. I now look around at my fellow riders and think about what “we” are all doing, how fast “we” are all going, and the aesthetic of it all. On reflection, my early feeling of separation was also likely somewhat reinforced by the instructors past well-meant words about us – the class- here and how, working and performing together as one (unlike those on the sofa at home), and the contrasting reality of my performance.

Although subjectivities are clearly at play in the above situation, these do not necessarily oppose fundamental metaphysical contentions, well established in the philosophy of movement and kinetic materialism, that objects emerge into existence as a result of their speeds. Specifically, as a result of their velocities, decelerations and accelerations as variants on a plane of immanence (Deleuze, 2005; Nail, 2019). And indeed, whether occurring phenomenologically or metaphysically, subjectively or pre-subjectively, individually or collectively, the establishment of objects by their relative positions and speeds will become all the more significant in the future. In a world - as noted earlier and as will be explored further later - composed of ever more numerous, complex and networked objects possessing ever greater movements at ever greater speeds.

### Theoretical foundation IV: speeds create rates of happening

Much contemporary process philosophy has been underpinned by the understanding that the fundamental basis of existence – of space and time - is actual occasions or events. According to this ontology, all change and motion in the world is the difference that results from a succession of discontinuous transitions caused by a succession of discontinuous events, the latter typically of variable duration and spacing (the result being an uneven ‘strobe becoming’). More recent philosophies of motion instead argue that the fundamental basis of existence – of space and time – is not events but continual motion - i.e. the flow of matter - that always both pre-exits and creates events (Nail, 2019). As alluded to earlier, flows of matter folding themselves up in particular ways and patterns, and completing flow cycles, to form temporary events (Nail, 2019). Either way though, whether they are primary or secondary features of the world, events happen, are important, and happen at/with certain speeds. In numerous social contexts, considering or reporting the rate of happening of events - their time taken – is more common than considering or reporting pure speeds/rates of motion within them (as noted previously for time geography (e.g. Cachinho and Paiva, 2021)). In everyday life, for example, we tend to report the (clock) time taken to go shopping, or the time taken to complete a jog, or the time taken for a computer to compete a task, rather than report the average speed of the shopper in the supermarket aisle, or the average speed of the runner on the road, or the average speed of incoming data moving through a processor (Armstrong, 2000; Bale, 2004). The same common approach held in the field. GA, for example, reflects on the emphasis placed on the completion of a ride:

**Recreational trail cycling GA:** This week we took five minutes less to complete the overall ride; to get all the bikes and riders to the end together as a group. We beat the twenty-five minute threshold.

Of course, the rate of happening of events is always determined by the speed of the constituent objects involved in creating them. In this sense the rate of happening of events is the speed of objects in motion in functional combinations and arrangements. Every movement of an object involves that object moving into some new relation which serves a larger process and outcome of some form (Andrews et al 2022; Nail, 2019). Hence, moving objects possess function and reactivity and are critical to agency and change in the world (Massumi, 2002). Being relatively abstract, one had to think about these ideas carefully in the field, but they were nonetheless reflected in the activities undertaken. GA, for example, reflects generally:

**Spinning GA:** Thinking today about how speed changes my world… Faster speeds whilst spinning has led to my being fitter quicker, some marginal weight loss, and ultimately to a feeling of general wellbeing and moving faster. In turn, I can now finish tasks, such as gardening, a little quicker

Subjectivities arise with most instances of rates of happening, as they arise with rates of motion, and are one basis for a human sense of temporality. Based on foundational philosophical work on perception and temporality - from Bergson’s ‘duration’, to Whitehead’s ‘specious present’, to Deleuze’s ‘crystals of time’– is the understanding that time is created for the moving human body by its constant relations with other bodies, objects and images (Bergson, 1889). On the more intense experiential ends, for humans, the passage of time might seem to speed up (compact) or slow down (stretch). This as they might feel related variations, such as future events being ‘close’ or ‘far off’, present events ‘dragging on’ or ‘passing quickly’, and past events being ‘recent’ or ‘long over’ (Andrews, 2021). Such assessments and feelings were certainly reflected in the field. MG, for example, recalls the different subjective experiences of going to, and returning from, work whist cycling:

**Commuter cycling MG:** Getting to work feels like it goes more quickly than does coming home, despite the distance being identical (and in fact coming home being ever-so-slightly uphill). There’s something in perception about purpose – my ‘to’ journey filled with promise of tasks and potential progress on that endless to-do list, whilst my ‘home’ journey spent reflecting on what was done, what remains, and what chores or responsibilities are looming.

Such assessments and feelings are experiential departures of speed from clock time, existing on the same forward continuum but not attuned to its exactness and its unvarying regularity. They are departures that certainly differ between bodies of different capacities, needs and alignments. More generally, there are those who argue that, in our increasingly goal-orientated, structurally-diverse, technologically-mediated, multi-experiential society - with flexible and individualised working and social lives and powerful commodities that create their own worlds -that ‘event time’ increasingly takes precedent over clock time, whether it be subjectively experienced or objectively judged (Adkins, 2009).

### Theoretical foundation V: speeds occur in all other material expressions/textures of space

Not only is speed a texture of space in its own right, as suggested previously, it is present in all other textures of space – rhythms, momentums, positions, spacings, shapes, lines of action, repetitions and infections (Andrews et al 2022). This is because these all involve movements so necessarily involve speed (Massumi, 2002; Morton, 2016). With regard to lines of action, rhythms and momentums, they might involve a high volume or intensity of objects in a given location moving at complementary speeds in complementary directions in a common production of some type (this contrasting with a situation involving a high volume or intensity of objects in a given location moving at different or oppositional speeds and directions, which limits the emergence of lines of action, rhythms and momentums). Both situations were reflected in the field. GA, for example, discusses the complementary lines of action and rhythms in spinning:

**Spinning GA:** The bikes we use are unusual in that, although stationary, they can lean/tilt. This replicates cornering in outdoor cycling; a deeper lean replicating a faster or tighter corner. Leaning is an important part of each session in terms of entertainment, exercises and building core strength. These leans are short, subtle but powerful lines of action. As a group we lean to the right towards the reception area, then left towards the fire exit, back and forth. The sessions also involve music. We change tempo with the changing tempo and structure of the accompanying music. Slower verses involve slower pedaling, faster pre-choruses involve building speed, fast choruses involve fast sprints. Certain slower songs chug along consistently at mid-tempo. They accompany high resistance. Today, the song’s lyric repeated “breathe in, breath out” and so we did, pedaling one half rotation with each breath.

CP, on the other hand, recalls the interrupted momentums of road cycling:

**Fitness road cycling CP:** I’m new to road bikes and still grappling with an unfamiliar gear set up. Mistakes are common and my rides are still punctuated by moments of frustration where I lose rhythm. My legs spin uncontrollably as I speed downhill in too low a gear. Or the wrong gear on inclines causes me to push hard against the resistance found, desperately trying to generate speed and zig-zagging my steering to keep balance. The mechanisms clunk and whir as I nudge right and left gear levers impatiently, causing the chain to leap back and forth up and down the cogs.

Meanwhile, affects/infections work ‘deep’ within and throughout space. All objects/bodies not only affect and are affected - contributing to their becoming and that of space - all are composed of smaller objects that affect and are affected, and all are part of larger objects that affect and are affected (Anderson, 2005; Massumi, 2002). Moreover, positive affection might involve objects in a location becoming harmonized or complementary in their speeds and directions, energizing one another in a common production of some type. This contrasts with negative affections that might involve objects in a location becoming disharmonized or uncomplimentary in their speeds and directions, working against each other and deenergizing one another (Anderson, 2005; Massumi, 2002). Such understandings were recognisable in the field experiences. GA, for example recalls how positive affection in spinning is often assisted by narrative, and CP recalls how negative affection emerges from cogs running at different speeds, the noises they make and what they induce:

**Spinning GA:** The instructor leads by energetic example but her instruction occurs throughout the session to keep the speed and energy high and us working together; “pop it up”, “pick it up”. Her positive encouragement and feedback includes “you’re doing great” “you’re killin it”. Some of her words might be more meaningful and hence more fully internalized “if it doesn’t challenge you, it doesn’t change you” “you are not failing if you are moving in the right direction” “you can feel the burn, but we are all going through it together”. I also notice that she refers to us in collective terms; “Monday” (or whatever day it is) and “class” being common. All sessions include a slower “your intention track”. It doesn’t seem to kill the energy, rather is seems to prepare us for future effort. During that track, with a very Deleuzian framing, the instructor asks us “how are you emerging? who are you evolving from and into?”

**Fitness road cycling CP:** When I make mistakes, the clunking sound of the cogs stirs up a residual nervousness in me. This originates from the time I made such a mess of things that the chain dropped off the cogs completely and the pedals – with my feet clipped firmly in place – locked instantly. Terrifying.

In sum, only all together do the material expressions/textures of space create the continuing moment (Andrews and Duff, 2020; Andrews et al 2022). None are ever absent, just further to or from the fore in terms of subjective experience and physical effectiveness. Indeed, it is not that space is a product of events and flows, with certain expressions/textures then individually and periodically proceeding from it. Rather, space is a product of events and flows with all expressions/textures as simultaneous omnipresent proceedings (Thrift, 2003).

### Theoretical foundation VI: the accelerating world is engaged at relational speeds

As described earlier, speed defines much of the current era. Fixated on the performances, functions, feels and aesthetics that speed elicits, humans have enrolled nonhumans in creating a society where things move ever faster (Duffy, 2009; Virilio, 1986); this now a general context for any entity’s path in the world. Ultimately, however because of the ways in which commercial and political interests and contemporary cultures prioritise and fashion space, multitemporal society and human lives have emerged. On one level, as noted, this expresses as certain people and places being included and others excluded from the faster world. On another level, this expresses as situations where humans moving through space unavoidably have to navigate the different speeds of events taking place; different paces of life (some of these events being brief and fleeting, whilst others constituting longer sections of lifecourses (Levine, 1997; Paiva et al 2017; Cachinho and Paiva, 2021)). On a final level, multitemporal society and lives expresses as situations where humans attempt occasionally to put themselves in positions to ‘speed up’ and/or ‘slow down’ mentally and physically; to change, enhance or escape predominant speeds of events taking place. Movement activity is often judged to facilitate such experiences; it often relatively fast yet escapist. These realities were reflected in the field experiences (they present a more general confirmation of theoretical foundation III - speed is known through relative positions). Whilst CP reflects on a slower ride, GA reflects on the ‘time out’ of a spinning class:

**Fitness road cycling CP:** It’s the weekend and I purposefully leave my watch at home as I head off on a known circuit. Desperate to take time out from the constant need for efficiency and timeliness, today I simply want to “pootle”. Removing my watch is symbolic of that. I still take my phone, joking with my partner that it’s my puncture repair kit. Throughout my ride I am conscious of its presence in my pocket and the strong pull to take it out and check the time.

**Spinning GA:** My own life – like many peoples’ lives – can be fast paced. Travelling to and from work, moving quickly from task to task. But spinning happens at the end of the day when that rush is over. The instructor seems to know this. The session begins with their narrative about “nothing existing for this time outside these walls – our problems all locked out”. There is feeling of slowing down for a moment, even though we are just about to speed up. Speed up we do but in a stress-relieving as opposed to stress-creating way. It’s a different form of speed that speeds our troubles away.

‘Fitting cycling in’ was however a consistent challenge for the researchers, as their busy working and family lives had to be prioritised. In response, whilst the dedication of regular days and timeslots for cycling helped, opportunities were also taken for cycling whenever and however they presented themselves. In this regard, MG notes:

**Commuter cycling MG:** A friend commented on my carrying my helmet into work today, something about “fitting exercise into daily routines”, and how commuting “works well for this”. While she’s not wrong, exercise not why I cycle commute – which, many days, makes the daily routine (daycare drop-offs & pick-ups) trickier, logistically. I do it to go outside; to ensure I get to enjoy a little piece of a beautiful day by moving through it

Despite cycling being an escape from the fast pace of life, it became clear that ‘fast-capitalism’ (Agger, 2015; Andrews and Duff, 2020b) was very much at work in it, with-companies rapidly collecting data from us, synthesizing it, and producing commercial responses. GA comments on what seems to be happening in terms of the monitoring of the technologies he uses and of his ride data:

**Recreational trail cycling GA:** Its obvious that our GPS’ and Fibits are monitored and our data mined. This because, soon after rides, we seem to have fitness and lifestyle products advertised to us through google and various social media. It seems uncanny how accurate the targeting it is, and I must say that its easy to get enrolled and want to purchase things that might make the rides easier, more comfortable, more productive and ultimately faster.

Such monitoring and advertising continued long after the study period ended, and continued to the time of writing, as we carried on cycling in our regular lives. We are clearly recognized, captured and monitored by companies, and there are seemingly few limits to the speeds at which new fitness and lifestyle products are suggested to us across media platforms. This no matter how much we try to disengage with the computational and commercial processes ongoing around us.

### Summary and future considerations

As we noted, the current theoretical orientation of human geography has been fruitful for the discipline. At its best, its view of space as vital, material and forever emerging, has shed light on the energetic geographies of the world, and produced work that is captivating, exciting and even hopeful (Philo, 2017). This has been partly achieved through a focus on the expressions/textures of space, but with speed as an occasional empirical focus rather than a consistent theoretical one. In terms of what we achieved in the current paper, through our ‘theory first, data second’ reconnaissance, we established some foundational understandings and principles of speed, then spatially animated them as empirical realities, as speed was shown to be a fundamental constitutive dimension of life. In sum these understandings are, speed can be represented and affective as a scalar quantity (a key practice in a technologically-mediated world); all objects possess speed and affect other speeds (a truth that no object is excluded); speeds and objects are known through relative positions and speeds (there is only one baseline speed in the world – the speed of light - all others are relative); speeds create rates of happening (a common consideration and experience of speed); speeds occur in all textures/expressions of space (the most fundamental texture/expression of them all); the accelerating world is engaged at relational speeds (humans and nonhumans do not always move at the same speed as larger orders). In particular, we showed that, although some theoretical understandings of speed might seem abstract, they transfer quite freely into everyday life situations, practices and experiences. Indeed, speed, and these understandings, are there in the subtle and in the obvious, in the notable and in the mundane, in the essential and in the inert. Certainly, we only touched upon each understanding, and the full scope of each remains to be explored. Moreover, in cycling we chose a fairly obviously ‘speedy’ activity to animate speed through; one that was relatively short in duration and limited in its scale and scope (at least for each occurrence). So it is our hope that scholars might follow our lead and move towards animating speed in many other empirical contexts; including speeds occurring across large and complex systems, speeds occurring over longer timeframes, and speeds that relate to other areas of economic, political, social and cultural life. The theoretical understandings that we animated in this paper could be useful as baseline, helping scholars to find and comprehend speed, its generation and consequences, across multiple domains.

Beyond the foundational theory we engaged with, and the empirical extensions just noted, there is however other work to be conducted on speed by geographers, particularly on how forms and roles of speed temporalize and shape the present conditions of life; on the economic and political forces that create and stabilize these speeds, and their consequences for social and natural systems (Duclos et al. 2017; Rosa, 2003; 2010). Moreover, on the involvement in these speeds of numerous entities each with particular movements, roles and relationships - some included and others excluded, some beneficiaries and others price payers - all connected via entangled practices (Creswell, 2010; Massey, 1994). Such a call for research is certainly consistent with the case that has been advanced for developing ‘dromology’; the study of speed as an academic discipline (Virilio, 1986). This a case based, for the most part, on understanding and engaging the specific empirical reality of the increasing speed of the world (as we have engaged with partly ourselves). But given the varied and omnipresent speed that exists in the world and as a texture of space irrespective of such acceleration (as we have also shown), there is a valid argument that dromology could be more broadly theoretically and empirically based (Andrews et al 2022). Hence, as geographers, we need to think about how we wish to frame our studies of speed in relation to this wider academic project; outside of it, or within it actively helping to broaden and deepen it.

With this in mind, key theoretical avenues for future inquiry that emerge from our study, might be grouped into four categories. These are certainly substantive categories, yet they merely reflect speed’s centrality in all life and the diversity of that life. (i) *Speed in/of humans and nonhumans*. Recent scholarship in human geography moves away from the view that humans are autonomous agents, towards the much more relational view that humans are co-dependent on nonhumans which also possess the capacity for agency (Andrews and Rishworth, 2023; Castree and Nash, 2006; Falcon, 2023). We need then think about the forms, roles and registers of speeds in human and nonhuman existences (each through engagements with the other). How speed exists in basic manifestations, movements and expressions of humans and nonhumans, and involves non-contemplative, pre-reflective and fully-conscious practices and registers. (ii) *Speed in social productions*. Recent scholarship in human geography moves away from the view that the world’s productions are human achievements, towards the view that they are outcomes of relations of humans and nonhumans (Andrews and Rishworth, 2023; Castree and Nash, 2006; Falcon, 2023). We need then to think about the role speed plays in these co-productions; how multiple biological, material and technological entities use speed together as they are at work in co-productions. This includes attention, not only to positive social productions (i.e. those that are ideal for humans and nonhumans) that may attract, enhance, add to, amplify, build, free and enthral, but also to negative social productions (i.e. those that are less than ideal for humans and nonhumans) that may repel, diminish, extract, silence, break, restrict and disenchant (Philo, 2017). The latter resulting in what Philo terms ‘less-than-human geographies’, that are more-than-human in their construction, but less-than-human in their humanity. (iii) *Speed in nature destructions*. Recent scholarship in human geography moves away from the view that humans and nature are separate categories (the former taking from and doing things to the latter), towards a view of them as one in (ontological) form, co-involved in processes and sharing some common outcomes (there being a hope here that humans might better realise and modify their role in the planet’s future) (Andrews and Rishworth, 2023; Castree and Nash, 2006; Falcon, 2023). We need then to think about the role that the relative speeds of biological, material and technological entities play in such destructions as pollution, environmental and species destruction and climate change, as well as in potential mitigation strategies and practices.

A final category of inquiry is a general research category; (iv) *Speed in geographical knowledge production and translation*. First and foremost, we need as scholars to develop a specific wonderment (Woodyer and Geoghegan, 2013) with speed. This an inquisitiveness and openness to see and feel speed at work in the world as we go about our daily lives and consider new areas of research. This so that speed emerges and sticks at the fore of our geographical imaginations (hopefully the current paper will help in drawing this out). It is wonderment that, at the same time, would equally appreciate our navigation of speeds in our own research and working lives. How different forms of speed temporalize the conditions of our production of research knowledge (Berg and Seeber, 2016; Duclos et al, 2017) as we attempt to meet deadlines and adhere to numerous timeframes, and as we attempt to reconcile the resulting speeds with those of our non-working lives. After developing such wonderment, many opportunities might arise in the academic enterprise of studying speed in the areas of theory, methods and disciplines. With regard to theory, we need to think more about how different theoretical traditions inside and outside human geography might understand and articulate particular aspects of speed and hence contribute to the overall conceptual understanding. And, in turn, about how engaging with speed might contribute to these theoretical traditions. With regard to methods, we need to think practically about how speed might be better isolated, recoded and animated as a texture of space in empirical research in ways that find the sweet spot between falling back on representation and deadening speed, and becoming giddy on speed’s freedom and aesthetics (McCormack, 2002). Moreover, about how speed might be used politically in experimental, creative methods to directly act into and change the world in the unfolding moment (Thrift, 2008). Finally, with regard to disciplines, we need to think about how our various geographical subdisciplines and other domains of academic concentration, might inform and advance ideas on speed as it occurs in the world. And, in turn, about how these subdisciplines and domains might be informed and advanced through engaging with speed. As we illustrated, speed is far more than a variable to be measured or facet or experience to be noted, it is a constitutive dimension of all entities and spaces (Duclos et al. 2017). Hence a deeper understanding and appreciation of speed should play dividends across human geography.

## Figures and Tables

**Table 1:** Longstanding visions of space and their limits

Theoretical Tradition	Key Vision	Limits
**Newtonian *‘absolute space’*** Has underpinned much spatial science since the mid twentieth century (particularly early studies)	Space as characterless, uniform and neutral; preexisting and independent from objects.	Space is not an empty container to the world which lies within it; it is not reducible to a void.
**Euclidean/Cartesian ‘*geometrical*** ***space’*** Has also underpinned much spatial science and also quantitative research more broadly	Space possesses three dimensions along which coordinates can be designated	Space is not the distance or shape between coordinates. It is not reducible to smooth abstract mathematical measurements (albeit that mathematical algorithms and computational systems increasingly shape social spaces)
**Marxist ideas on the ‘*capitalist*** ***production of space’*** Have underpinned much (post)radical geography in the last five decades.	Because capitalism requires a ‘spatial fix’ – i.e. the acquirement of new space in an addictive sense to avoid overaccumulation, and the use of space in anchoring sense to operate - it fashions space	Space is not primarily driven by, and constituted of, industrial practices and the social divisions they create. It is not reducible to relations of production.
**Phenomenological ideas on** **‘*known space’*** Have underpinned much humanistic and social constructionist geography in the past four decades.	Space is known through human embodiment (the experience of presence and involvement) and subsequent thought processes and meaning attributions (albeit that certain humanists - such as Tuan - consider space to be more abstract and less open to such processes than place).	Space is not primarily imagined and represented linguistically and textually into being something. It is not ultimately reducible to human engagement and subjectivity
**Certain poststucturalist thinking** **on *power-generated space*** Have underpinned cultural geography in recent years.	Most notably Foucauldian explanations regard space as being the result of the exercising of predominant forms of power (whilst the exercising of power is also facilitated by space)	Space is not primarily disciplined into being through control and constraint. Such a vison leaches out space’s freedom and vitality, and, beyond notions of ‘resistance’, does not fully appreciate the extent and ways that, as Thrift suggests, just as it plays with humans, humans play with it

**Table 2:** Emerging understandings of space in the new human geography

Consideration	Understanding
Existence of space	Space is not something that occurs on its own. It is not a fabric in and of itself; it is dependent on objects, an emergent effect of their relationality. Space comes into being only through relationships between objects of positionality, causality and change.
Kinetics of space	Because all objects – from those extensive and complex, to those minute and simple - are in continual motion to some degree, the object-relations that create space are in constant flux. Ultimately then, space is constituted of a network of relational material flows (each with function) and itself flows.
Reproduction of space	Because in any region of space objects arise and cease, move in and out, and form impermeant assemblages of particular character, space is itself ontogenetic; in a never-ending process of being (re)made
Productive capacities of space	In the constant (re)making of space, there exists all possibilities and actualities of the world’s trajectories. All objects, systems and outcomes that make the world in all its diversity, emerge as dynamic temporary unities within, and expressions of, assemblages of objects and relations in space.
Processual orders in space	Space creates it own mechanisms from the ground up that emerge within assemblages. A Deleuzian reading is that assemblages that facilitate particular mechanisms (such as forms of power or identity), assemble physically through functional and affective forces (rather than through the mechanism itself). Mechanisms arise only as a stratified outcome of assemblages and their relations
Excessiveness of space	On one level, space is ontologically more than the sum its parts (its constituent entities) in terms of its qualities, productive capacities and experience. On another level, counter to the general trend of increasing disorder in the universe, certain regions of (social) space tend to move themselves, at least initially, to greater order and complexity. This excessiveness of space evades formal measurement and quantification (though it is often achieved and recognised through/as space’s expressions/textures).
Character and registering of space	The changing relational positions and functions of objects give space particular *forms* (such as sounds and shapes) and make it highly varied. Thereafter, space is relative and particular to entities. Entities register space uniquely from their specific locations during the time they are present in them. And those that change locations register ‘new’ spaces over time from successive locations.

**Table 3:** Speed as a measure, variable and quality in human geography

Level and Description	Examples
**Level One:** speed used in academic studies, just as it is everyday common language	Speed is part of describing movements, expansions, spreads and rapidity in phenomenon, ranging from humans to pathogens, vehicles, housing, data, markets, policy, development and beyond (e.g. Banister, 2011; Givoni and Banister, 2012; Haynes et al 2006; Huang et al 2021; Kamel Boulous and Geraghty, 2020; Lawson et al 2019; Quddus, 2013)
**Level Two**: speed is more central in empirical analysis	Speed helps bring principles of acceleration and velocity into the quantitative mapping and modelling of spatial relationships (Janelle, 1968; Isard, 1971; Isard and Liossatos, 1975).
**Level Three**: speed in an implicit quality of space	The role, experience and practice of speed is traceable in varied studies of human movements in new mobilities geographies, and sports and fitness geographies (Andrews, 2017; Bissell, 2007).
**Level Four**: speed is a key concept and quality of a field	Speed figures centrally in time geography as a quantity that contributes to the awareness of movements through space and time at different scales, and their time taken as events. Here speed is one value of entities (the others being direction and distance) applied in the space-time path and prism diagrams common to the field (Ellegård, 2018; Hägerstrand, 1970; Miller, 2005; Thrift 1977).

**Table 4:** Critiques of time-space compression

Area of critique	Description
Increased standardization	The standardization of relations and encounters under time-space compression has led to increased homogenization and reduced potential for the emergence of local, novel and unique events and things (Massey, 1991; 1994) (albeit, as Massey suggests, being continually reconstructed from outside, the events and things that result are still diverse, dynamic and changing).
Spatial unevenness	Compression processes have been uneven through time and geographically, such as from country to country, and urban to rural (May and Thrift, 2001)
Inclusion and exclusion	Whilst certain entities (humans, other biological organisms, materials, technologies and places), are excluded from time-space compression processes, others are included and ‘used’ by them. Compression processes impact on different entities in different ways and to different extents (Warf, 2011).
Spaces have also expanded	The spaces of events have not only compressed under time-space compression, in certain contexts the spaces of events have also expanded (such as new global spaces opening up). Similarly the times taken for events have not only compressed under time-space compression, in certain contexts the times taken for events have also lengthened (such as certain economic activities now being undertaken twenty-four hours a day) (May and Thrift, 2001).
World (space) has expanded	Over recent years, science has realized that the overall durations and extents – i.e. times and spaces - of species, civilizations and the universe are longer and larger than once thought (so the overall time and space map of the world has enlarged) (May and Thrift, 2001).

**Table 5:** Facets and features of contemporary ‘fast capitalism’

Fact/Feature	Overview
Conquering of space and time with speed	Conquering space and time with speed is related directly to time-space compression, and is key to capitalism’s ongoing accumulation (Harvey, 1989). In doing this, the local – and its richness and uniqueness - is surplus to requirements, apart from its role in providing particular character sources of production/supply and consumption/demand (Luke and Tuathail, 1998; Warf, 2011).
Facilitation by neoliberal ‘free’- market policy and deregulation	Free markets allow accelerating modes of disruption, innovation, change, exchange and accumulation. Globalization processes are key here, as they allow new minimally-regulated labor and consumer markets to be opened up (Munck, 2002). However this expansion comes with substantial costs, such as destruction of natural environments, exploitation of labour, unethical practice, data breaches, cyclical economic crises, social inequalities and discontent (Agger, 2015; Rose, 2017).
Connecting to consumers in subtle yet powerful and targeted ways	Fast capitalism reaches out to, and draws in, consumers far more effectively and comprehensively than past forms of ever did with far more suggestive power. Trading in immediate sensory, affective and emotional experience, it exploits consumers’ propensities, rapidly and temporarily fulfilling their desires and cravings for immediate gratification (Sampson, 2016; Thrift, 2008). This engineering of addictive consumption, has also been termed ‘narco-capitalism’ (Virilio, 1995).
Knowing consumers comprehensively and responding rapidly	Fast capitalism knows consumers and potential consumers far more than earlier forms ever did. This knowing is facilitated by technology which offers the ability to harvest data on consumer behaviors, synthesize this data in almost zero-time, and enact rapid market responses (Suarez-Villa, 2012; Thrift, 2005).

## References

[R1] Adkins L (2009). Sociological futures: from clock time to event time. Sociological research online.

[R2] Agger B (2015). Speeding up fast capitalism: Cultures, jobs, families, schools, bodies.

[R3] Ahn K, Rakha H, Trani A, Van Aerde M (2002). Estimating vehicle fuel consumption and emissions based on instantaneous speed and acceleration levels. Journal of transportation engineering.

[R4] Ameel L, Tani S (2012). Parkour: Creating loose spaces?. Geografiska Annaler: Series B, Human Geography.

[R5] Anderson B (2014). Encountering affect: Capacities, apparatuses, conditions.

[R6] Anderson B, Tolia-Kelly D (2004). Matter (s) in social and cultural geography. Geoforum.

[R7] Anderson B, Kearnes M, McFarlane C, Swanton D (2012). On assemblages and geography. Dialogues in human geography.

[R8] Andrews GJ (2017). From post-game to play-by-play: Animating sports movement-space. Progress in human geography.

[R9] Andrews GJ (2020). Health geographies III: More-than-representational pushes and expressions. Progress in Human Geography.

[R10] Andrews GJ (2021). Bios and arrows: On time in health geographies. Geography Compass.

[R11] Andrews GJ, Duff C (2020a). ‘Whole onflow’, the productive event: an articulation through health. Social Science & Medicine.

[R12] Andrews GJ, Duff C (2020b). Enrolling bodies, feasting on space: How wellbeing serves the forms and flows of a new capitalism. Wellbeing, Space and Society.

[R13] Andrews GJ, Duff C, Woodward K, Gorman R (2022). Speed and space: rates of motion in health and wellbeing. Wellbeing, Space & Society.

[R14] Andrews GJ, Rishworth A (2023). New theoretical terrains in geographies of wellbeing: Key questions of the posthumanist turn. Wellbeing, Space and Society.

[R15] Angrosino MV (2007). Naturalistic observation.

[R16] Armstrong MP (2000). Geography and Computational Science. Annals of the Association of American Geographers.

[R17] Badiou A (2005). Being and Event.

[R18] Bale J (2004). Running cultures: Racing in time and space.

[R19] Bale J, Sang J (1996). Kenyan running: Movement culture, geography and global change.

[R20] Banister D (2011). The trilogy of distance, speed and time. Journal of Transport Geography.

[R21] Barad K (2007). Meeting the universe halfway: Quantum physics and the entanglement of matter and meaning.

[R22] Berg M, Seeber BK (2016). The slow professor: Challenging the culture of speed in the academy.

[R23] Bergson H (1889). Time and Free Will (trans. Pogson, FL).

[R24] Bissell D (2007). Animating suspension: Waiting for mobilities. Mobilities.

[R25] Bissell D (2013). Habit displaced: The disruption of skilful performance. Geographical Research.

[R26] Bissell D, Vannini P, Jensen OB (2017). Intensities of mobility: kinetic energy, commotion and qualities of supercommuting. Mobilities.

[R27] Braun V, Clarke V (2021). Thematic analysis: A practical guide. Sage.

[R28] Bridge G (2020). Habit, experience and environment: a pragmatist perspective. Environment and Planning D: Society and Space.

[R29] Brown KM (2017). The haptic pleasures of ground-feel: The role of textured terrain in motivating regular exercise. Health & Place.

[R30] Brown K, Spinney J (2010). Catching a glimpse: The value of video in evoking, understanding and representing the practice of cycling. Mobile methodologies.

[R31] Buttimer A (1976). Grasping the dynamism of lifeworld. Annals of the association of American geographers.

[R32] Cachinho H, Paiva D (2021). The enactment of fast and slow time regimes by urban retail and consumer services. Annals of the American Association of Geographers.

[R33] Cadman L, Kitchin R, Thrift N (2009). International Encyclopedia of Human Geography.

[R34] Castree N, Nash C (2006). Posthuman geographies. Social and Cultural Geography.

[R35] Conradson D, Bissel D, Fuller G (2011). Stillness in a Mobile World.

[R36] Cook M, Edensor T (2017). Cycling through dark space: Apprehending landscape otherwise. Mobilities.

[R37] Cook S, Shaw J, Simpson P (2016). Jography: Exploring meanings, experiences and spatialities of recreational road-running. Mobilities.

[R38] Crang M, Hassan R, Purser R (2007). 24/7: Time and Temporality in the Network Society, Stanford.

[R39] Cresswell T (2010). Towards a politics of mobility. Environment and planning D: society and space.

[R40] Cresswell T, Merriman P (2011). Geographies of mobilities: Practices, spaces, subjects.

[R41] Crewe L, Bruzzi S, Gibson PC (2000). Fashion cultures revisited: Theories, explorations and analysis.

[R42] DeLanda M (2016). Assemblage theory.

[R43] Deleuze G (1994). Difference and repetition.

[R44] Deleuze G, Fraser M, Greco M (2005). The Body.

[R45] Deleuze G, Guattari F (1988). A thousand plateaus: Capitalism and schizophrenia.

[R46] Deleuze G, Guattari F (1994). What is philosophy?.

[R47] Denton GG (1966). A subjective scale of speed when driving a motor vehicle. Ergonomics.

[R48] Denton GG (1980). The influence of visual pattern on perceived speed. Perception.

[R49] DeVerteuil G (2022). Slow scholarship, the slow city, and counter-visual practices. Environment and Planning F.

[R50] Dewsbury J-D (2000). Performativity and the Event: Enacting a Philosophy of Difference. Environment and Planning D: Society and Space.

[R51] Dewsbury JD, Bissell D (2015). Habit geographies: the perilous zones in the life of the individual. cultural geographies.

[R52] Dodgshon RA (1999). Human geography at the end of time? Some thoughts on the notion of time—space compression. Environment and Planning D: Society and Space.

[R53] Doel MA (1996). A hundred thousand lines of flight: a machinic introduction to the nomad thought and scrumpled geography of Gilles Deleuze and Félix Guattari. Environment and planning D: society and space.

[R54] Doel MA (2000). Crang M and Thrift Thinking space.

[R55] Duclos V, Criado TS, Nguyen VK (2017). Speed: An Introduction. Cultural Anthropology.

[R56] Duffy E (2009). The speed handbook: Velocity, pleasure, modernism.

[R57] Edensor T (2010). Walking in rhythms: Place, regulation, style and the flow of experience. Visual Studies.

[R58] Edensor T (2012). Geographies of rhythm: nature, place, mobilities and bodies.

[R59] Edensor T (2000). Walking in the British countryside: Reflexivity, embodied practices and ways to escape. Body & Society.

[R60] Einstein A (1905). Zur deutschen Literatur für Viola da Gamba im 16. und 17, Jahrhundert 2, 1.

[R61] Elert G (2022). The physics hypertextbook.

[R62] Ellegård K (2018). Thinking time geography: Concepts, methods and applications.

[R63] Evers C (2009). ‘The Point’: surfing, geography and a sensual life of men and masculinity on the Gold Coast, Australia. Social & Cultural Geography.

[R64] Falcon J (2023). Toward a critical posthuman geography. cultural geographies.

[R65] Foley R (2015). Swimming in Ireland: Immersions in therapeutic blue space. Health & Place.

[R66] Foley R (2017). Swimming as an accretive practice in healthy blue space. Emotion, Space and Society.

[R67] Givoni M, Banister D (2012). Speed: the less important element of the High-Speed Train. Journal of Transport Geography.

[R68] Glass MR, Rose-Redwood R (2014). Performativity, politics, and the production of social space.

[R69] Green K (2011). It hurts so it is real: Sensing the seduction of mixed martial arts. Social & Cultural Geography.

[R70] Hägerstrand T (1970). What about people in regional science.

[R71] Harman G (2018). Speculative realism: an introduction.

[R72] Harvey D (1989). The condition of postmodernity.

[R73] Harvey D (1996). Justice, nature and the geography of difference.

[R74] Harvey D (1982). The limits to capital.

[R75] Harvey D (2001). Globalization and the “spatial fix”. geographische revue: Zeitschrift für Literatur und Diskussion.

[R76] Haynes R, Jones AP, Sauerzapf V, Zhao H (2006). Validation of travel times to hospital estimated by GIS. International Journal of Health Geographics.

[R77] Huang Q, Zhang H, van Vliet J, Ren Q, Wang RY, Du S, He C (2021). Patterns and distributions of urban expansion in global watersheds. Earth’s Future.

[R78] Ireland AV, Finnegan-John J, Hubbard G, Scanlon K, Kyle RG (2019). Walking groups for women with breast cancer: mobilising therapeutic assemblages of walk, talk and place. Social Science & Medicine.

[R79] Isard W (1971). Spatial interaction analysis: Some Suggestive thoughts from general relativity physics. Papers of the Regional Science Association.

[R80] Isard W, Liossatos P (1975). Parallels from physics for space-time development models: Part I. Regional Science and Urban Economics.

[R81] Janelle DG (1968). Central place development in a time-space framework. The Professional Geographer.

[R82] Jones M (2009). Phase space: geography, relational thinking, and beyond. Progress in human geography.

[R83] Jones P (2012). Sensory indiscipline and affect: a study of commuter cycling. Social & Cultural Geography.

[R84] Kamel Boulos MN, Geraghty EM (2020). Geographical tracking and mapping of coronavirus disease COVID-19/severe acute respiratory syndrome coronavirus 2 (SARS-CoV-2) epidemic and associated events around the world: how 21st century GIS technologies are supporting the global fight against outbreaks and epidemics. International journal of health geographics.

[R85] Kanngieser A (2012). A sonic geography of voice: Towards an affective politics. Progress in Human Geography.

[R86] Kitchin R, Dodge M (2005). Code and the Transduction of Space. Annals of the Association of American geographers.

[R87] Latham A (2015). The history of a habit: jogging as a palliative to sedentariness in 1960s America. cultural geographies.

[R88] Latham A, McCormack D, Hall T, Hubbard P, Short JR (2008). The Sage Companion to The City.

[R89] Lawson B, Potter A, Pil FK, Holweg M (2019). Supply chain disruptions: the influence of industry and geography on firm reaction speed. International Journal of Operations & Production Management.

[R90] Lea J, Cadman L, Philo C (2015). Changing the habits of a lifetime? Mindfulness meditation and habitual geographies. cultural geographies.

[R91] Larsen J (2018). Commuting, exercise and sport: an ethnography of long-distance bike commuting. Social & Cultural Geography.

[R92] Lefebvre H (2004). Rhythmanalysis: Space, time and everyday life.

[R93] Leibniz GW, Alexander HG (1956). Mr. Leibniz’ third paper 1716. In H G Alexander ed The Leibniz – Clark Correspondence.

[R94] Levine RN (1997). A geography of time: On tempo, culture, and the pace of life.

[R95] Levine R (2005). A geography of busyness. Social Research: An International Quarterly.

[R96] Lorimer H (2012). Surfaces and slopes. Performance Research.

[R97] Luke TW, Tuathail GÓ (1998). An Unruly World?: Globalization, Governance, and Geography.

[R98] Luke T, Tuathail GO, Crang M, Thrift N (2002). Thinking space.

[R99] Macpherson H (2009). Articulating blind touch: Thinking through the feet. The Senses and Society.

[R100] Macpherson H (2008). “I don’t know why they call it the Lake District they might as well call it the rock district!” The workings of humour and laughter in research with members of visually impaired walking groups. Environment and Planning D: Society and Space.

[R101] Malpas J (2012). Putting space in place: Philosophical topography and relational geography. Environment and Planning D: Society and Space.

[R102] Marston SA, Jones JP, Woodward K (2005). Human geography without scale. Transactions of the institute of British geographers.

[R103] Massey D (1992). 1992: Politics and space/time. New Left Review.

[R104] Massey D (1994). Space, Place, and Gender.

[R105] Massey DB (2005). For Space. SAGE.

[R106] Massumi B (2002). Parables for the Virtual: Movement, Affect, Sensation.

[R107] May J, Thrift N (2001). Timespace: geographies of temporality.

[R108] McCormack DP (2002). A paper with an interest in rhythm. Geoforum.

[R109] McCormack DP (2004). Cultural geographies in practice: Drawing out the lines of the event. Cultural geographies.

[R110] McCormack DP (2005). Diagramming practice and performance. Environment and planning D: society and space.

[R111] McCormack DP (2013). Refrains for moving bodies: Experience and experiment in affective spaces.

[R112] McCormack DP (2017). The circumstances of post-phenomenological life worlds. Transactions of the Institute of British Geographers.

[R113] Mels T (2016). Reanimating places: A geography of rhythms.

[R114] Merriman P (2012). Human geography without time-space 1. Transactions of the institute of British Geographers.

[R115] Merriman P, Jones M, Olsson G, Sheppard E, Thrift N, Tuan YF (2012). Space and spatiality in theory. Dialogues in Human Geography.

[R116] Merriman P (2022). Space.

[R117] Merriman P (2023). Crystallising places: Towards geographies of ontogenesis and individuation. Progress in Human Geography.

[R118] Meyer M (2024). A calculative cosmography: Geodesy and absolute space. Environment and Planning F.

[R119] Middleton J (2009). ‘Stepping in time’: walking, time, and space in the city. Environment and planning A.

[R120] Middleton J (2010). Sense and the city: exploring the embodied geographies of urban walking. Social & cultural geography.

[R121] Middleton J (2011). “I’m on autopilot, I just follow the route”: Exploring the habits, routines, and decision-making practices of everyday urban mobilities. Environment and Planning A.

[R122] Miller HJ (2005). A measurement theory for time geography. Geographical analysis.

[R123] Morton T (2016). Moving, still: introduction to Gaffney N Stillness: a book of photographs FoAM Publishing and.

[R124] Munck R (2002). Globalisation and labour: the new’Great Transformation’.

[R125] Nail T (2019). Being and motion.

[R126] Nail T (2021). Theory of the Earth.

[R127] Nash C (2000). Performativity in practice: some recent work in cultural geography. Progress in Human Geography.

[R128] Norman JF, Ross HE, Hawkes LM, Long JR (2003). Aging and the perception of speed. Perception.

[R129] Paiva D (2018). Sonic geographies: Themes, concepts, and deaf spots. Geography Compass.

[R130] Paiva D, Cachinho H, Barata-Salgueiro T (2017). The pace of life and temporal resources in a neighborhood of an edge city. Time & Society.

[R131] Parkes DN, Thrift N (1975). Timing space and spacing time. Environment and Planning A.

[R132] Paterson M (2009). Haptic geographies: ethnography, haptic knowledges and sensuous dispositions. Progress in human geography.

[R133] Pavlidis A, Fullagar S (2015). The pain and pleasure of roller derby: Thinking through affect and subjectification. International Journal of Cultural Studies.

[R134] Pfister H (2004). Newton’s First Law Revisited. Foundations of Physics Letters.

[R135] Philo C (2012). A ‘new Foucault’with lively implications–or ‘the crawfish advances sideways’. Transactions of the Institute of British Geographers.

[R136] Philo C (2017). Less-than-human geographies. Political Geography.

[R137] Pickles J (1985). Phenomenology, science and geography: spatiality and the human sciences.

[R138] Pile S (2010). Emotions and affect in recent human geography. Transactions of the Institute of British Geographers.

[R139] Pred RJ (2005). Onflow: Dynamics of consciousness and experience.

[R140] Quddus M (2013). Exploring the relationship between average speed, speed variation, and accident rates using spatial statistical models and GIS. Journal of Transportation Safety & Security.

[R141] Raco M, Durrant D, Livingstone N (2018). Slow cities, urban politics and the temporalities of planning: Lessons from London. Environment and Planning C: Politics and Space.

[R142] Rietveld P, Shefer D (1998). Speed choice, speed variance, and speed limits: A second-best instrument to correct for road transport externalities. Journal of Transport Economics and Policy.

[R143] Rodaway P (2002). Sensuous geographies: body, sense and place.

[R144] Rosa H (2003). Social acceleration: ethical and political consequences of a desynchronized high–speed society. Constellations.

[R145] Rosa H (2005). The speed of global flows and the pace of democratic politics. New Political Science.

[R146] Rosa H (2010). High-speed society: Social acceleration, power, and modernity.

[R147] Rose G, Massey D, Allen J, Sarre P (1999). Performing spaceIn Human geography today.

[R148] Rose N (2017). Still ‘like birds on the wire’? Freedom after neoliberalism. Economy and Society.

[R149] Sampson T (2016). Various joyful encounters with the dystopias of affective capitalism. Ephemera: Theory and Politics in Organization.

[R150] Searle A, Turnbull J, Lorimer J (2021). After the anthropause: Lockdown lessons for more-than-human geographies. The Geographical Journal.

[R151] Shaw IG (2012). Towards an evental geography. Progress in Human Geography.

[R152] Schwanen T (2007). Matter (s) of interest: Artefacts, spacing and timing. Geografiska Annaler: Series B, Human Geography.

[R153] Schwanen T, Banister D, Anable J (2012). Rethinking habits and their role in behaviour change: the case of low-carbon mobility. Journal of Transport Geography.

[R154] Sheppard E, Barnes T (1990). The capitalist space economy: Geographical analysis after Ricardo, Marx and Sraffa.

[R155] Simandan D (2016). Proximity, subjectivity, and space: Rethinking distance in human geography. Geoforum.

[R156] Simpson P (2017a). Spacing the subject: Thinking subjectivity after non-representational theory. Geography Compass.

[R157] Simpson P (2017b). A sense of the cycling environment: Felt experiences of infrastructure and atmospheres. Environment and Planning A: Economy and Space.

[R158] Simpson P (2019). Elemental mobilities: atmospheres, matter and cycling amid the weather-world. Social & Cultural Geography.

[R159] Spinney J (2006). A place of sense: a kinaesthetic ethnography of cyclists on Mont Ventoux. Environment and Planning D: Society and Space.

[R160] Spinney J (2007). Horton et al Cycling and society.

[R161] Spinney J (2009). Cycling the city: Movement, meaning and method. Geography compass.

[R162] Suarez-Villa L (2012). Technocapitalism: A critical perspective on technological innovation and corporatism.

[R163] Thorpe H, Rinehart R (2010). Alternative sport and affect: Non-representational theory examined. Sport in Society.

[R164] Thrift N (1977). Time and theory in human geography: part II. Progress in Human Geography.

[R165] Thrift N (1996). Spatial formations.

[R166] Thrift N, Clifford N, Holloway S, Rice SP, Valentine G (2003). Key concepts in geography.

[R167] Thrift N (2004). Movement-space: The changing domain of thinking resulting from the development of new kinds of spatial awareness. Economy and Society.

[R168] Thrift N (2005). Knowing capitalism.

[R169] Thrift N, Crampton JW, Elden S (2007). Space, knowledge and power: Foucault and geography Ashgate.

[R170] Thrift N (2008). Non-representational theory: Space, politics, affect.

[R171] Tomlinson J (2007). The culture of speed: The coming of immediacy.

[R172] Vannini P, Adey P, Bissell D, Hannam K, Merriman P, Sheller M (2014). The Handbook of Mobilities.

[R173] Vannini P (2015). Non-representational methodologies.

[R174] Vannini P, Taggart J (2015). Solar energy, bad weather days, and the temporalities of slower homes. Cultural Geographies.

[R175] Virilio P (1986). Speed and Politics: An Essay on Dromology.

[R176] Virilio P (1995). Speed and information: Cyberspace alarm!. ctheory.

[R177] Virilio P (2005). Desert screen: War at the speed of light.

[R178] Waitt G, Buchanan I (2023). Velomobilities: Cycling geographies and well-being. Geography Compass.

[R179] Waitt G, Cook L (2007). Leaving nothing but ripples on the water: performing ecotourism natures. Social & Cultural Geography.

[R180] Wajcman J, Dodd N (2016). The sociology of speed: Digital, organizational, and social temporalities.

[R181] Warf B (2008). Time-space compression: Historical geographies.

[R182] Warf B (2011). Teaching time–space compression. Journal of Geography in Higher Education.

[R183] Whatmore SJ (2009). Mapping knowledge controversies: science, democracy and the redistribution of expertise. Progress in Human Geography.

[R184] Whatmore SJ (2013). Earthly powers and affective environments: An ontological politics of flood risk. Theory, Culture & Society.

[R185] Williams N, Patchett M, Lapworth A, Roberts T, Keating T (2019). Practising post-humanism in geographical research. Transactions of the Institute of British Geographers.

[R186] Wilson HF (2017). On geography and encounter: Bodies, borders, and difference. Progress in Human Geography.

[R187] Woodyer T, Geoghegan H (2013). (Re) enchanting geography? The nature of being critical and the character of critique in human geography. Progress in Human Geography.

[R188] Wylie J (2005). A single day’s walking: narrating self and landscape on the South West Coast Path. Transactions of the institute of British Geographers.

[R189] Wylie J, Anderson B, Harrison P (2010). Taking Place: Non-Representational Theories and Geography.

